# Alzheimer’s disease hypothesis and related therapies

**DOI:** 10.1186/s40035-018-0107-y

**Published:** 2018-01-30

**Authors:** Xiaoguang Du, Xinyi Wang, Meiyu Geng

**Affiliations:** 1 Shanghai GreenValley Pharmaceutical Co., Ltd., 421 Newton Road, Shanghai, 201203 People’s Republic of China; 2 State Key Laboratory of Drug Research, Shanghai Institute of Materia Medica, Chinese Academy of Sciences, 555 Zu Chong Zhi Road, Shanghai, 201203 People’s Republic of China

**Keywords:** Alzheimer’s disease - complicated disease - anti-AD drugs, Hypothesis about AD

## Abstract

Alzheimer’s disease (AD) is a progressive neurodegenerative disorder and the most common cause for dementia. There are many hypotheses about AD, including abnormal deposit of amyloid β (Aβ) protein in the extracellular spaces of neurons, formation of twisted fibers of tau proteins inside neurons, cholinergic neuron damage, inflammation, oxidative stress, etc., and many anti-AD drugs based on these hypotheses have been developed. In this review, we will discuss the existing and emerging hypothesis and related therapies.

## Background

Alzheimer’s disease (AD) is a progressive neurodegenerative disorder, which is the most common cause for dementia and imposes immense suffering on patients and their families. According to the World Alzheimer Report 2016, there are currently about 46.8 million people suffering with AD worldwide. The ageing of world population will further compound this problem and lead to a steep increase in the number of AD patients. The numbers of AD patients are expected to double nearly every 20 years, and thereby the population of AD will reach 74.7 million in 2030 and 131.5 million in 2050 [1]. AD has become the third major cause of disability and death for the elderly, only after cardiovascular and cerebrovascular diseases and malignant tumors.

However, only five drugs have been approved by the FDA to treat AD over the past hundred years since the first AD patient was diagnosed. Not only that, these approved drugs including cholinesterase inhibitors, N-methyl-D-aspartate (NMDA) receptor antagonist or their combination usually provide temporary and incomplete symptomatic relief accompanied with severe side effects. The marginal benefits were unable to slow the progression of AD. Thus, developing drugs for more effective AD treatment is in urgent need.

### Current hypothesis about AD and anti-AD drug development

AD is a complicated disease involving many factors. Due to the complexity of human brains, the lack of reasonable animal models and research tools, the detailed pathogenesis of AD is still unclear so far. Many hypotheses about AD have been developed, including amyloid β (Aβ), Tau, cholinergic neuron damage and oxidative stress, inflammation, etc. Thus, many efforts have been done to develop anti-AD drugs based on these hypotheses.

#### Aβ cascade hypothesis

Extracellular deposits of Aβ peptides as senile plaques, intraneuronal neurofibrillary tangles (NFTs), and large-scale neuronal loss were the main pathological features of AD. Thus, Aβ peptides have long been viewed as a potential target for AD which dominated new drug research during the past twenty years [2]. The most direct strategy in anti-Aβ therapy is to reduce Aβ production by targeting β- and γ-secretase [3]. Safety issues are the overriding problem. For targeting γ-secretase, undesirable side effects are inevitable due to its physiological substrates, eg. the Notch signaling protein [4–7], which is essential in normal biological process. Similarily, targeting β-secretase is also challenged for the side effects such as blindness and the large catalytic pocket [8]. More importantly, in sporadic AD cases, the majority of AD patients do not necessarily have over-producted amyloid precursor protein. Besides, Aβ isoforms could also serve as endogenous positive regulators for neurotransmitter release at hippocampal synapses [9]. Thus, inhibiting Aβ production may encounter many challenges.

Aβ clearance by immunotherapy is the alternative choice. For active Aβ-immunotherapy, although the first active AD vaccine (AN1792) developed by ELAN showed some beneficial effects such as less cognitive decline, it was suspended owing to serious side effect, or meningoencephalitis [10–12]. Also, the passive immunotherapy did not do much better than active immunotherapy. Several antibodies targeting Aβ have failed in clinical trials, including bapineuzumab (Pfizer/Johnson & Johnson) [13, 14], Crenezumab (Genentech) [15, 16], solanezumab (Eli Lilly) [16–18] and ponezumab (Johnson & Johnson /Pfizer) [19–21]. In addition, although passive immunotherapy could overcome some problems of active immunotherapy, there were still inevitable side effects such as amyloid-related imaging abnormalities [22]. Likewise, the small molecule Aβ binder scyllo-inositol [23] and tramiprosate [24–26] also failed in clinical trials. These failures even cast more doubts on the Aβ theory [27]. Actually, the strategy of targeting only a single functional subregion of Aβ may partly account for these failures [27, 28]. Furthermore, immunotherapy may influence the human immune system, which might cause beneficial or detrimental consequence (such as side effects). However, every cloud has a silver lining. A phase Ib trial of aducanumab (Biogen) showed a positive correlation between brain Aβ levels and disease exacerbation as measured by Clinical Dementia Rating [29–31]. Even the failed phase III EXPEDITION3 trial of solanezumab (Eli Lilly) still demonstrated better performance in Clinical Dementia Rating Sum of Boxes and beneficial impacts on Mini-Mental State Examination and Activities of Daily Living [17, 18, 32, 33]. Thus, despite all kinds of problems, immunotherapy may still be the better approach to modify the extent of neurodegeneration in AD currently [34].

In fact, the original amyloid cascade hypothesis was that “Aβ is the causative agent in Alzheimer’s Disease pathology, and that neurofibrillary tangles, cell loss, vascular damage, and dementia follow as a direct result of this deposition” [35]. After decades of research, although the bulk of data still supports a role for Aβ as the primary initiator of the complex pathogenic cascade in AD, more and more evidences indicate that Aβ acts as a trigger in the early disease process and appears to be necessary but not sufficient in the late stage of AD [36]. Especially, recent rapid progresses in understanding on toxic amyloid assembly and Aβ metabolism associated systemic abnormalities will provide fresh impetus and new opportunities for this interesting approach [37].

#### Tau hypothesis

Neurofibrillary tangles, another intracellular hallmark of AD, are composed of tau. Tau is a microtubule-associated protein working as scaffolding proteins that are enriched in axons. In pathological conditions, tau aggregation will impair axons of neurons and thus cause neurodegeneration. After numerous failures of Aβ-targeting drugs for AD, more interests are turning to explore the therapeutic potential of targeting tau, particularly as studies of biomarkers suggest that tau pathology is more closely linked to the progression of AD [38].

Tau undergoes many modifications, including phosphorylation, arginine monomethylation, lysine acetylation, lysine monomethylation, lysine dimethylation, lysine ubiquitylation and serine.

O-linked N-acetylglucosamine (O-GlcNAc) modification [39]. Under pathological conditions, increasing of tau hyperphosphorylation will render the protein aggregation-proned, reduce its affinity for microtubules, and thereby influence neuronal plasticity. Consequently, strategies to target tau involve blocking of tau aggregation, utilizing tau vaccinations, stabilizing microtubules, manipulating kinases and phosphatases that govern tau modifications. However, most of these efforts have failed in clinical trials. For Tau aggregation blockers, TRx0237 failed to show treatment benefits in phase III trials [40]. As for vaccinations, tau-targeted active vaccines (ACI35 and AADvac-1) and passive vaccines (RG6100 and ABBv-8E12) are currently in phase I and II clinical trials [41, 42]. Intravenous immunoglobulin (IVIG), the only passive vaccine in phase III clinical trials, failed to meet the primary end points in patients with mild-to-moderate AD [42]. Other tau-targeting strategies for AD, including stabilizing microtubules and manipulating kinases and phosphatases, have just been tested in preclinical studies.

In general, tau-targeting therapies remain challenging because of incomplete understanding of AD, lack of robust and sensitive biomarkers for diagnosis and response-monitoring, and the obstruction of blood-brain barrier.

#### Inflammation hypothesis

Reactive gliosis and neuroinflammation are hallmarks of AD. Microglia-related pathways were considered to be central to AD risk and pathogenesis, as supported by emerging genetic and transcriptomic studies [43–47]. Increasing evidence demonstrate that microglia emerges as central players in AD. In very early stage, microglia, TREM2 and complement system are responsible for synaptic pruning [48, 49]. The processes of activity-dependent and long-term synaptic plasticity are the common and fundamental cellular underpinning of learning and memory which may manifest as influence on long term potential [50]. Following that, reactive microglia and astrocytes will surround amyloid plaques and secrete numerous pro-inflammatory cytokines. These events are regarded as an early, prime mover in AD evolution. However, non-steroid anti-inflammatory drugs (NSAIDs) did not show enough benefits in clinic. This is because that the relationship between innate immunity and AD pathogenesis is complex, and the immune response can be either deleterious or beneficial depending on the context [47, 51, 52]. However, the new observations that PD-1 immune checkpoint blockade reduces the pathology of AD and improves memory in mouse models of AD [53–55] give us a direction of future researches.

The recent advances in our understanding of the mechanism underlying microglia dysfunction in pruning, regulating plasticity, and neurogenesis are opening up possibilities for new opportunities of AD therapeutic interventions and diagnosis [56, 57]. Targeting these aberrant microglial functions and thereby returning homeostasis may yield novel paradigms for AD therapies. However, given the complexity and diverse functions of microglia in health and disease, there is a crucial need for new biomarkers reflecting the function of specific microglias [52, 58].

#### Cholinergic and oxidative stress hypothesis

Acetylcholine (ACh) is an important neurotransmitter used by cholinergic neurons, which has been involved in critical physiological processes, such as attention, learning, memory, stress response, wakefulness and sleep, and sensory information [59–63]. Cholinergic neurons damage was considered to be a critical pathological change that correlated with cognitive impairment in AD. Thus, cholinergic hypothesis was firstly tested with cholinesterase inhibitors in AD treatment. Tacrine, a cholinesterase inhibitor, was the first anti-AD drug available in clinic [64–66] although it was withdrawn from the market in 2012 due to severe side effects. Although inhibiting cholinesterase is a symptomatic relief treatment with marginal benefits, it is currently the most available clinical treatment which gives desperate AD patients a glimmer of hope. For other neurotransmitter dysfunction, such as Dopamine and 5-hydroxytryptamine, there are some studies about them, but not much as acetylcholine in AD.

Oxidative stress is considered to play an important role in the pathogenesis of AD. Especially, the brain utilizes more oxygen than other tissues and undergoes mitochondrial respiration, which increases the potential for ROS exposure. In fact, AD is highly associated with cellular oxidative stress, including augmentation of protein oxidation, protein nitration, glycoloxidation and lipid peroxidation as well as accumulation of Aβ, for Aβ can also induce oxidative stress [67–73]. Thus, the treatment with anti-oxidant compounds would provide protection against oxidative stress and Aβ toxicity in theory. However, oxidative stress is only a single feature of AD, so antioxidant strategy was challenged for its potency to stop the progression of AD and thus it is proposed as a portion of combination therapy [74, 75].

### Glucose hypometabolism

Glucose hypometabolism is the early pathogenic event in the prodromal phase of AD, and associated with cognitive and functional decline. Early therapeutic intervention before the irreversible degeneration has become a consensus in AD treatment. Thus, alleviation of glucose hypometabolism was emerged as an attractive strategy of AD treatment. However, most of these therapeutic strategies are targeting mitochondria and bioenergetics, which have shown promise at the preclinical stage but without success in clinical trials [76, 77]. Although no strategies are available to alleviate glucose hypometabolism in clinical, glucose metabolism brain imaging such as ^18^FDG-PET (Positron emision tomography with 2-deoxy-2-fluorine-18-fluoro-D-glucose) has become a valuable indicator for diagnosis of neurodegenerative diseases that cause dementia, including AD [78].

Up to now, there’re no effective treatments for changing the course of AD. Confronting these difficulties, we should get deeper understandings about these hypotheses, and meanwhile we should renovate our knowledge about AD and develop new hypothesis.

### New pathway to AD

AD is conventionally regarded as a central nervous system (CNS) disorder. However, increasing experimental, epidemiological and clinical evidences have suggested that manifestations of AD extend beyond the brain. Most notably, research over the past few years reveals that the gut microbiome (GMB) has a profound impact on the formation of the blood-brain barrier, myelination, neurogenesis, and microglia maturation [79–84]. In particular, results from germ-free animals and animals exposed to pathogenic microbial infections, antibiotics, probiotics, or fecal microbiota transplantation showed that gut microbiota modulates many aspects of animal behaviors, suggesting a role for the gut microbiota in host cognition or AD-related pathogenesis [85–88]. The underlying mechanisms of gut microbiota influencing brain involve the communication through immune system, the endocrine system, the vague nerve, and the bacteria-derived metabolites.

#### Immune pathway

The intestinal mucosal lymphoid tissue contains 70% ~ 80% of the immune cells in the whole body, and is considered to be the largest and most important human immune organs. It is also the first line of host defense against pathogens. The human gut contains a large, diverse and dynamic enteric microbiota, including more than 100 trillion microorganisms from at least 1000 distinct species. There’s a complex relationship between intestinal mucosal immune system and intestinal microbiota. Thus, gut microbiota induced immunomodulation is emerging as an important pathway that influences AD [89].

Gut microbiota can influence brain through immune system in several ways. Firstly, intestinal microbiome can induce cytokines secretion, which enter the circulatory system, pass through blood brain barrier, and directly affect the brain function. For instance, perivascular macrophages and cerebral small vessel epithelial cells can receive the intestinal microbiome produced IL-1 signal and affect central nervous system. Also, gut microbes can activate Toll-like receptors of the brain immune cells (such as microglia) through microbes associated molecular patterns (MAMP). MAMPs can either directly bind to intestinal epithelial cells or infiltrate to the intestine lamina propria to activate lymphocytes, promoting the release of pro-inflammatory cytokines, which further cause subsequent inflammation in brain. Secondly, gut microbes can produce metabolites such as short-chain-fatty acids (SCFAs), gamma-aminobutyric acid (GABA) and 5-HT precursors, which could also travel to the brain via circulatory systems or signal through intestinal epithelials to produce cytokines or neurotransmitters that activate vagus nerve. Thirdly, gut microbes can activate enteroendocrine cells to produce 5-HT, which affect the brain through neuroimmune pathways.

In addition to changing the functions of the immune system, such as through secretion of inflammatory factors or anti-inflammatory factors, intestinal microbiome can also affect the development and composition of immune system. For example, in germ-free mice, isolated lymphoid follicles in gut associated lymphoid tissue are unable to mature, and lymphocytes that are able to secrete IgA in the intestinal epithelium decreased [89–92]. For immune system in brain, the deletion of gut microbiota in germ-free mice have global influence on the cell proportions and maturation of microglia in the brain, and thus affect the properties and phenotype of microglia, as compared to conventionally colonized controls [93]. Similar results were obtained in antibiotic treated mice. Other research also demonstrates that the number of T regulatory cells and T helper lymphocytes (T helper 17, Th17) are significantly reduced in the germ free mouse, indicating the regulatory effects of intestinal microbiome on T cell composition, while microbiome tansplant to germ free mice can modify these variations and restore normal immune function [94, 95]. All these modulations of gut microbiota may have direct and indirect effects on AD development and progression.

#### Endocrine pathway and the vagus nerve

The gut is also the largest endocrine organ in the body. Gut microbiota can regulate secretion of many hormones from intestinal endocrine cells, such as corticosterone and adrenal hormones, and thus establish the information exchange between the intestines and the brain. For example, the intestinal microbiome can affect the secretion of serotonin and regulate brain emotional activities [96, 97]; intestinal microbial metabolism can also produce a variety of neurotransmitters, such as dopamine, GABA, acetylcholine and melatonin, which are transmitted to central nervous system through the vagus nerve [98]. Besides transporting these signal substances, the vagus nerve itself plays an important role in inflammation and depression [99]. The vagus nerve can influence the gastrointestinal tract, orchestrate the complex interactions between central and peripheral neural control mechanisms [100]. The stimulation of vagus nerve is able to regulate mood, and the immune system, suggesting the therapeutic potential of vagus nerve modulation to attenuate the pathophysiological changes and restore homeostasis [98–103].

#### Bacteria-derived metabolites

Generation of essential nutrients for host physiology, such as vitamins and other cofactors, is an important physiological function of the gut microbiota [104]. The metabolites of microbiome, such as SCFAs including acetate, butyrate, and propionate, are able to modulate peripheral and central pathologic processes [105]. For example, butyrate is effective in reducing inflammation and pain. Once in the brain, acetate is able to alter the level of the neurotransmitters glutamate, glutamine, and GABA, as well as increases anorectic neuropeptide expression [106]. In addition, the gut microbiota can secrete large amounts of amyloids and lipopolysaccharides, which might contribute to the modulation of signaling pathways, the production of proinflammatory cytokines associated with AD pathogenesis and Aβ deposition [107–109].

In fact, microbiota-gut-brain axis has been established and a disturbed gut microbiota has been incriminated in many neurodegenerative diseases in animal and translational models. In theory, a role for the microbiota-gut-brain axis is highly plausible. However, the theoretical basis for the use of microbiota-directed therapies in neurodegenerative disorders still needs supports from high-quality clinical trials [110]. To date, only a few studies directly focused on the gut microbiota and AD [111, 112], and studies on AD patients is particullarly deficient. A recent research from human showed an increase in the abundance of a pro-inflammatory GMB taxon and a reduction in the abundance of an anti-inflammatory taxon are possibly associated with a peripheral inflammatory state in patients with cognitive impairment and brain amyloidosis. It is important for the research of gut microbiota and AD. However, further investigations are still necessary to explore the possible causal relation between GMB-related inflammation and amyloidosis [111]. The comprehensive understanding of these underlying mechanisms may provide new insights into these novel therapeutic strategies for AD. In particular, based on the gut microbiota hypothesis, Chinese traditional medicine and probiotic bacteria may play a more important role in therapy [113].

## Conclusions

Nowadays, new technologies are making it possible to get to know enough pathologic details of disease. More importantly, scientists are beginning to treat AD as a systemic disease and they are paying more attention to the correlation between brain and other organs [47, 89, 114]. Perhaps, for complicated disease such as AD, researches and therapies should be based on the principle that combined reductionism with holism, and great efforts should be made to search the fundamental laws of AD by means of multi-scale modeling and efficient numeric assessment. Maybe, just like Chinese traditional medicine [115], combination treatments or systematic therapy will be a final way out.

## Abbreviations

AD: Alzheimer’s disease
Aβ: Amyloid β
CNS: Central nervous system
CSF1R: Colony stimulating factor 1 receptor
GABA: Gamma-aminobutyric acid
IVIG: Intravenous immunoglobulin
MAMP: Microbes associated molecular patterns
NFTs: Neurofibrillary tangles
NMDA: N-methyl-D-aspartate
NSAIDs: Non-steroid anti-inflammatory drugs
O-GlcNAc: O-linked N-acetylglucosamine
SCFAs: Short-chain-fatty acids
Th17: T helper lymphocytes 17
